# Assessing young adults' menopause knowledge to increase understanding of symptoms and help improve quality of life for women going through menopause; a student survey

**DOI:** 10.1186/s12905-023-02641-4

**Published:** 2023-09-15

**Authors:** Vaishvi Patel, Sue Ross, Beate C. Sydora

**Affiliations:** Department of Obstetrics and Gynecology, Faculty of Medicine & Dentistry, Women and Children’s Health Research Institute, University of Alberta, 626-1 Community Service Centre, Royal Alexandra Hospital, 10240 Kingsway Ave, Edmonton, T5H-3V9 Canada

**Keywords:** Menopause, Menopause symptoms, Menopause experience, Menopause knowledge, Quality of Life, Climacteric symptoms

## Abstract

**Background:**

Due to menopause being a largely invisible and under-discussed topic in wider society, women often deal with menopause-related complications on their own. Social support and awareness have been shown to reduce negative menopausal experiences; however, lack of menopause knowledge, particularly among younger people, may deter support for women suffering from menopause symptoms. This study aims to assess the level of knowledge young adults have on menopause to be able to create interventions that target knowledge gaps and increase understanding of women’s experiences and difficulties during their menopause transition.

**Methods:**

We created an electronic questionnaire based on menopause literature and guidelines from Menopause Societies. It was pilot-tested on young people in the target group age (*n* = 14; 7 male and 7 female), menopause clinicians (*n* = 5), and women experiencing menopause (*n* = 4). The final survey included questions on participant demographics, general menopause knowledge, and options to support menopause management and was distributed through university student newsletters. Responses over a two week period were collected anonymously. Descriptive statistics were applied to characterize participants, define menopause knowledge, and identify gaps. Chi-squared statistics was used for group comparison, and open questions were analyzed using qualitative content analysis.

**Results:**

Survey responses were collected from 828 students; the average age was 22.1 ± 5.1 and 83.6% were female. Participants belonged to all faculties and included students from a variety of family settings and living conditions. Knowledge questions revealed a good understanding of the basic menopause physiology for most respondents, but there were gaps in understanding of symptoms and symptom management. Female sex and personal connection to menopausal women had a positive effect on the degree of menopause knowledge. Both males and females reported increased knowledge confidence at the end of the survey.

**Conclusion:**

Our survey provides evidence that young adults of both sexes have a general baseline knowledge of menopause and its symptoms and are open to learning strategies to help support menopausal women. Our findings will assist in developing targeted educational resources to increase social support and awareness, reduce stigma and improve the quality of life for menopausal women, and help prepare younger women for their future menopause journey.

**Supplementary Information:**

The online version contains supplementary material available at 10.1186/s12905-023-02641-4.

## Background

Menopause is defined as the point in time when a woman has ceased having menstrual periods for 12 consecutive months, marking the end of her reproductive years. During menopause and its transition, estrogen deficiency can give rise to a variety of psychological, physical, and endocrine changes, including mood disorders, sleep disorders, sexual dysfunction, joint pain, body aches, hot flashes, and increased risk for the development of osteoporosis, and cardiovascular health issues [1–4].

Women's perception of menopause severity and symptoms varies widely and can be influenced by individual experiences related to cultural context, mental state, emotional health, and social support [3]. Studies have shown that women undergoing the menopause transition are psychologically more vulnerable and are at high risk for developing depression and mood disorders; these are attributed to several factors such as inadequate social support which is among the most important [5, 6]. Women are often reticent about menopause, causing it to be an unmentioned, largely invisible topic that is seldom discussed in wider society, leaving women to deal with menopause-related changes on their own. The stigma surrounding menopause, associations with mood changes, the shame of ageing, and the taboo about revealing menopausal symptoms, all contribute to hidden distress. Furthermore, the stigma from others’ reactions can become internalized, further decreasing quality of life. The result is a hidden public health disorder that can cause suffering for women as well as their family members [7–9].

Based on our research group’s previous work, menopausal women often report feeling anxious, frustrated, and alone because they feel their friends and family are not able to understand what they are experiencing and are not able to provide them with the support they need [8, 9]. Social support and education have been shown to reduce negative menopausal experiences and result in a higher quality of life for women during menopause [10, 11]. It has also been shown to reduce the risk of mental health disorders in women during the menopause transition [3]. Together these findings suggest that social support, including physical and emotional empathy, can be a simple, cost-effective, and sustainable way to help women through this transition, mitigating menopausal complications. Intervention programs designed to increase the awareness and education of menopausal women, as well as their spouses, have shown to be effective but there have not been interventions focused on improving the understanding of younger adults of both sexes about women's experience of menopause [2, 10]. Younger adults often consider menopause to be a topic of humor or fear. Educating young adult family members and relatives to better understand the topic of menopause will enable them to be supportive of menopausal women.

Therefore, this study was designed to assess young adults' basic knowledge about the topic of menopause, including its symptoms and management experience, in order to address their knowledge gaps through educational material. In this way, we hope to break the cycle of misunderstanding and create changes that will encourage more sympathetic responses to women experiencing menopause.

## Methods

To approach our research goal, we employed a cross sectional design in form of an electronic survey. The methodology and results of the survey were reported using published recommendations such as Checklist for Reporting of Survey Studies (CROSS) and Checklist for Reporting Results of Internet E-Surveys (CHERRIES) to ensure that this study was conducted in a reliable, reproducible, and transparent manner [12, 13].

The study was approved by the University of Alberta Health Research Ethics Board (Pro00108310).

### Design of an electronic survey

Our open survey was designed based on the menopause literature review and guidelines from the International Menopause Society (IMS) and the North American Menopause Society (NAMS) [14, 15]. We also sought inspiration from validated menopause questionnaires such as the menopause symptom rating scale (MSRS) [16] and the Menopause-specific Quality of Life (MENQOL) questionnaire [17–19], as well as a symptom severity questionnaire used to follow the progression of menopause symptoms in women treated at the two Menopause Outpatient Clinics in Edmonton, Alberta [20]. In addition, we considered common concerns raised by menopausal women in recently conducted focus groups at these outpatient clinics [8].

The electronic survey was created using the secure web application Research Electronic Data Capture (REDCap) [21] and included a demographic and menopause knowledge section. Within the demographics section, participant demographics such as age, sex, field of study, relationship with menopausal women and family structure and composition were asked. The menopause knowledge section included knowledge-based and opinion-based questions using multiple choice, sliding scale and short answers. Where applicable a non-response option was included.

### Pilot-testing of the survey

The first draft of the survey was pilot-tested to gather feedback on its usefulness, comprehensiveness, and adequateness before it was distributed. The survey was administered to (a) young adults (under 30 years of age) that are not current University of Alberta students (b) menopause clinic health professionals, and (c) menopausal women aged 50 and older. The participants were purposefully sampled and recruited through word-of-mouth of known contacts to the research team. The volunteers who agreed to participate received an e-mail containing the formal study information letter (including the consent process) and a unique URL to access a Google Form where they had an opportunity to review questions and provide feedback.

We received feedback from 23 individuals, 14 young adults (7 males, 7 females), 5 menopause health professionals, and 4 menopausal women aged 50 years and older. The survey draft was modified based on the feedback received. The final survey spanned 6 pages and took less than 20 min to complete.

Data collected from the pilot testing was not used in the analysis.

### Participant group and survey distribution

The survey was distributed in June 2020 through email to undergraduate and graduate students, enrolled at the University of Alberta through Undergraduate Digest Newsletter and Graduate Student Communications, respectively. The email contained a prompt along with a link to the survey. To partake in the survey, participants needed to meet the following inclusion criteria: currently enrolled at the University of Alberta and at least 18 years of age. A negative answer to these two questions terminated the survey. No specific exclusion criteria were applied. Completing the survey indicated consent.

While participation in the survey was voluntary, we employed a purposive sampling technique; we chose University of Alberta students as our target group as most students would belong within the younger adult demographic. In addition, this target group was most available, and conducting a survey was an appropriate method during the COVID-19 pandemic when no face-to-face interactions were permitted.

### Data acquisition

Study data was collected anonymously during a time spanning two weeks in June 2020 and stored in REDCap, accessible only to the researchers. Students were only able to partake in the survey once and their responses were saved every time an answer was entered. The participants were not able to alter their previous answers once they have proceeded to the next portion of the survey.

Upon completion of the survey, participants were redirected to another site where they could enter their name and email address to participate in a draw for a $ 50 (CAD) gift card. There was one random gift card draw for every 50 participants.

### Data analysis

For analysis, the data was exported into Microsoft Office Excel and descriptive analysis was applied. Questionnaires that were terminated early were not analyzed. Nine evidence-based knowledge questions were included with multiple choices or yes/no answers. Only one correct answer was available for each item. Each correct answer received one point; wrong answers were counted as 0 points. For each participant the points were summated, with the lowest score being 0 and the highest being 9; a higher score indicating more knowledge about menopause.

For statistical analysis of data comparison based on demographic factors, IBM-SPSS data analytics software version 28 was used. A chi-squared test of independence was performed to examine the relationship between sex and menopause knowledge levels and between having close contact with a woman in menopause and levels of menopause knowledge. Paired t-tests were used to compare means of self-assessed levels of menopause knowledge confidence at the start and at the end of the survey. Independent sample tests were used to compare these means between males and females and between participants who have close contact with a woman in menopause or do not. Qualitative content analysis was utilized to generate themes and summarize knowledge gaps from opinion-based and open-ended questions.

## Results

### Participant characteristics

Out of 929 returned surveys, 828 completed the demographic section. Of these, 76 were graduate and 752 were undergraduate students. Most participants were enrolled in the Faculty of Science (195, 23.6%), followed by Arts (160, 19.3%), and Engineering (93, 11.2%), which is representative of the size of the programs. The majority of participants were female (683, 82.5%) and identified as a woman (658, 79.5%). We received responses from students of all ethnic backgrounds with the most being Caucasian (432, 52.2%) followed by South Asian (123, 14.9%) and East Asian (111, 13.4%). Of all participants, 460 (55.6%) were single and 477 (57.7%) lived with their families. Only 8.7% of participants did not have some form of a relationship with someone currently in menopause while 81.6% of participants reported having a close contact with a woman in menopause. The average age of participants was 22.1 ± 5.1 (mean ± SD). Demographic details are provided in Additional file 1.

Respondents completed knowledge-based questions and questions on support and help for menopausal women. The complete questions and corresponding answer options can be found in Additional file 2.

### Menopause knowledge

An open-ended question “please describe what menopause is” was answered by 785 respondents (Table 1).

**Table 1:** Open answer question: “In a sentence, please describe what menopause is?”

Themes *N* = 785 responses	Topic ^a^	N [%]
Final menstruation	End of periods/menstrual cycles	524 [66.8]
	End of fertility	378 [48.2]
Hormonal changes	Change in hormones	229 [29.2]
	Reduction in Estrogen	35 [4.5]
Menopause timing	One year/12 months post last menstruation	17 [2.2]
Age at menopause	Described as post 40, between 40 and 60, or around age 50	85 [10.8]
Symptoms	General experience symptoms	85 [10.8]
	Hot Flashes	53 [6.8]
	Mental/Mood changes	41 [5.2]
	Sleep disturbance	4 [0.5]
Reduced QOL	Suffering/not feeling well	13 [1.7]
No/insufficient knowledge of menopause	“Don’t know” or “not sure”	6 [0.8]

^a^more than one answer is possible within topics

The majority (66.8%) described menopause as the time when menstrual periods stop and the end of fertility (48.2%), with a few mentioning that it starts 12 months post last menstrual period (2.2%). About a third (33.7%) acknowledge that this has to do with hormonal changes, and a few (4.5%) named estrogen and progesterone as the hormones involved. About a quarter (23.3%) mentioned that menopause relates to debilitating symptoms with a few mentioning prototypical menopause symptoms such as hot flashes and mood swings. Six admitted that they do not know, are not sure, or have no idea what menopause is.

Table 2 depicts the scores of participants on 9 knowledge-based questions. A chi-squared test of independence showed that there was a significant difference between menopause knowledge and sex (X^2^ (1, *N* = 780) = 11.9, *p* = 0.02); females demonstrated higher knowledge levels than males (Table 2A). There was also a significant difference between menopause knowledge level and whether the participants had close contact with a woman in menopause (X^2^ (1, *N* = 787) = 15.7, *p* = 0.003); participants with close contact to a woman undergoing menopause scored higher than those that did not (Table 2B).

**Table 2:** Level of menopause knowledge

**A) Knowledge-based questions (*n* = 9) by biological sex**			
**Number of correct answers**	**Female *N* = 651** **N [%]**	**Male *N* = 129** **N [%]**	**Prefer not to answer** **N [%]**
9	191 [29.3]	33 [25.6]	4 [57.1]
8	281 [43.2]	49 [38.0]	2 [28.6]
7	122 [18.7]	23 [17.8]	1 [14.3]
6	24 [3.7]	12 [9.3]	0 [0.0]
5 and less	33 [5.1]	12 [9.3]	0 [0.0]
**B) Knowledge-based questions (*n* = 9) by having close contact with a woman in menopause stage**			
**Number of correct answers**	**Close contact *N* = 648** **N [%]**	**No contact *N* = 139** **N [%]**	
9	198 [30.6]	30 [21.6]	
8	278 [42.9]	54 [38.8]	
7	116 [17.9]	30 [21.6]	
6	27 [4.2]	9 [6.5]	
5 and less	29 [4.5]	16 [11.5]	

### Knowledge about menopause symptoms

To assess knowledge of menopause symptoms, survey respondents were asked to check off all symptoms from a choice of 9 health issues that they believe are associated with menopause. 754 participants responded to this question. The majority correctly identified major menopause symptoms. Mood changes (728, 96.6%), fatigue (689, 91.4%), and hot flashes (682, 90.5%) were the three most picked answers by participants, while other symptoms such as depression, memory loss, aching joints and dry eyes were chosen by 29 to 70% of participants.

**Fig. 1:**
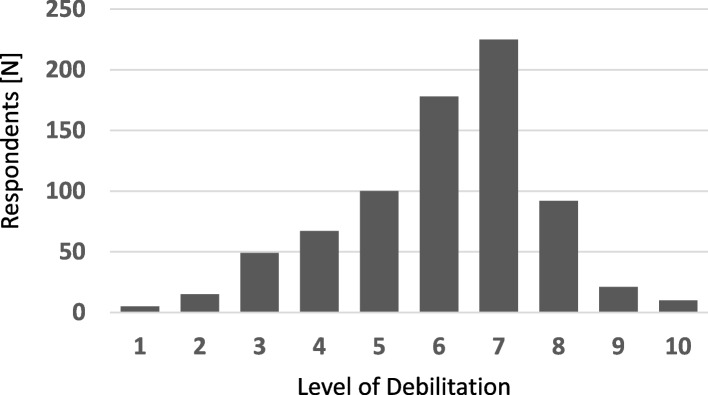
Rating of debilitation associated with menopause symptoms. Participants responded to the question “On a scale of 1–10, how debilitating do you think menopause symptoms are on average for women?” The number of responses at each level from 1 to 10 is shown. *N* = 762, median: 6, mean ± SD: 6.06 ± 1.67

Survey participants were also asked to score on a scale of 1 – 10, how debilitating they think menopause symptoms are on average for women. The scoring was done by 762 participants. On average the participants gave the symptom experience a score of 6 in terms of debilitation (Fig. 1).

### Menopause perception and opinion

To assess empathy and compassion towards menopausal women, participants were asked a variety of multiple-choice questions. The majority of participants believed that a lower socioeconomic status contributes to an earlier onset of menopause (62.6%) and that social isolation from friends and family is an ineffective way of managing menopausal symptoms (91.2%). Participants also indicated that providing social and emotional support is the most sustainable way to support a woman undergoing menopause (97.5%) (Table 3).

**Table 3:** Socioeconomic and empathy questions

Possible answers to questions	N [%]
**Can lower socioeconomic status contribute to an earlier onset of menopause?** ***N* = 763**	
Yes	478 [62.6]
No	285 [37.4]
**Which of the following is a likely scenario occurring due to menopause?** ***N* = 747**	
Janet forgetting where she put her phone	253 [33.9]
Amira slipping on a patch of ice in her driveway	21 [2.8]
Sumin starting to get oily skin and hair, and acne	459 [61.4]
Dyani having regular menses (periods)	14 [1.9]
**How can you help a woman undergoing menopause?** ***N* = 747**	
Tell her to get a grip	2 [0.3]
Tell her to take a break	5 [0.7]
Be kind and supportive	738 [98.8]
Leave her alone with her emotions	2 [0.3]
**Which of the following is the most sustainable way to support a woman undergoing menopause? *N* = 747**	
Buy her an AC unit	7 [0.9]
Provide social and emotional support	728 [97.5]
Tell her she will get over it	4 [0.5]
Keep your distance from her	1 [0.1]
Buy her a solo vacation to Paris	7 [0.9]
**Which of the following is an ineffective way of managing menopausal symptoms?** ***N* = 747**	
Exercising	21 [2.8]
Journaling every day	13 [1.7]
Social isolation from friends and family	681 [91.2]
Seeking medical help	27 [3.6]
Using a calendar to keep track of events	5 [0.7]

Participants were asked to rate their confidence and knowledge of menopause at the start and end of the survey on a scale from 1—10. Female participants gave themselves a higher score than their male counterparts. Similarly, those with relationship to a woman in menopause gave themselves a higher score than those without such a relationship. All groups of participants gave themselves a significantly higher score at the end of the survey compared to their pre-survey score (Table 4).

**Table 4:** Self confidence assessment of menopause knowledge

Respondents	N	Confidence level at start of survey [mean ± SD]	Confidence level at end of survey [mean ± SD]	Paired t-test at start versus end	Independent sample test between groups
All respondents	746	5.05 ± 2.06	5.32 ± 1.88	*p* < .001	na
Female	618	5.19 ± 1.96	5.43 ± 1.81	*p* < .001	at start: *p* < .001
Male	121	4.26 ± 2.40	4.76 ± 2.13	*p* = .006	at end: *p* < .001
Not disclosed	7	na	na	na	
Close contact to a woman in menopause	617	5.17 ± 1.99	5.42 ± 1.82	*p* = < .001	at start: *p* < .001
No close contact to a woman in menopause	129	4.47 ± 2.30	4.86 ± 2.07	*p* = .04	at end: *p* = .002

When asked the question “Would you be interested in learning more about menopause?”, most participants (674, 90.3%) indicated that they were interested in learning more about menopause with the majority interested in a website with educational resources (414, 55.5%), followed by a lecture delivered by a content expert (216, 29.0%) and physical printouts and brochures (44, 5.9%).

## Discussion

This study investigated university students’ knowledge and perception of menopause. We did not intend to provide education or investigate specific treatment options such as hormonal, non-hormonal drug/herbal and laser therapies. The survey findings suggest students have a basic knowledge of the physiology and the symptoms associated with menopause (Tables 1 and 2). In answering an open-ended question, most students mentioned that menopause was associated with hormonal changes and cessation of menstrual periods (Table 1). A few students also recognized hot flashes and mental/mood changes as major menopause symptoms. These two symptoms, in addition to fatigue, were also among the symptoms correctly chosen from a drop-down list as symptoms associated with menopause, indicating that students and young adults are aware of the stereotypical symptoms of menopause but may not be aware that menopause can encompass a much greater variety of symptoms including depression, memory loss, and dry eyes. This is consistent with other studies which show that even middle aged women and spouses of menopausal persons are not fully aware of the more hidden symptoms and experiences of menopause [22]. Nevertheless, based on the researcher-made scoring system of the questionnaire, most participants scored 7 or 8 out of 9, once again suggesting that students have a modest knowledge of menopause. Although these scores can be influenced by age, past experiences, and program of study, we found that both sex and close contact with a menopausal woman significantly impacted the scores. Females in comparison to males scored statistically higher and an even bigger difference was seen between participants who have close contact with a woman undergoing menopause compared to those without this contact, with the former scoring higher. These scores overlap with the participants’ perceived self-confidence scores on menopause knowledge, with females and those in contact with a menopausal person giving themselves a higher score than their counterparts (Table 4). In all cases, participants report a higher self-confidence score post-questionnaire indicating that thinking about menopause and being confronted with the topic is a learning process and boosts confidence in the topic.

The higher scores of female participants was an expected outcome as menopause is an almost universal stage in women’s health, but the increase in the menopause knowledge confidence score and the positive influence on scores through a close relationship to a menopausal person was an interesting and promising finding. This goes to show that individuals can be educated and informed about menopause and other potential conditions and that learning can be based on proximity and without a lived experience, similar to the spouses of those undergoing menopause, ultimately making young adults a teachable target demographic [23].

On a scale of 1 – 10, the participants rated the debilitation brought on by menopause on average as 6/10, indicating that there was a general recognition and awareness of the negative impact of menopause on quality of life (Fig. 1). Although none of the participants are likely experiencing menopause currently, they were able to apply their knowledge of the condition to understand the impact it has on quality of life. A similar result was observed in other scenario-based and awareness questions which highlighted participants’ empathy and thoughtfulness (Table 3). Studies have shown that the younger generation has the ability to consider others’ beliefs, express concern, and that their empathy leads to building social relationships [24, 25]. The aforementioned reasons, in addition to our findings that over 90% of participants indicated interest in learning more about menopause, make young adults an excellent demographic to target for an intervention.

Menopause, with its many complications, has adverse effects on the quality of life of women and places strain on their relationships, especially with their partners [9, 26]. A study by Currie and Moger which surveyed women 45 years of age or greater with menopause symptoms and their partners found that the physical and emotional effects of menopause affect women’s social life, professional lives, and relationships [27]. These women reported more frequent arguments with family members, feelings of isolation, negative impacts on sex life, missed work and decreased enjoyment in social interactions due to their symptoms. The stigma surrounding menopause further contributes to the decreased quality of life and prevents open communication with family members and friends and even deters women from getting help and advocating for themselves.

One potential solution is increasing social and emotional support for these women, which has been positively correlated with a decrease in the symptoms experienced and increases in health and prosocial behaviors and self‐esteem [3]. A study by Shariat-Moghani et al., in which husbands of postmenopausal women received educational programming regarding menopause and its complications, resulted in a decrease in negative menopause-related experiences by their spouses [10]. This study along with others confirms that increasing family awareness and support improves women’s experiences and psychological and physical health [11, 26, 28]. Another approach is to educate the pre-and peri- menopausal women themselves as most have limited knowledge about menopause, its symptoms, and management and often are interested to learn more about the topic [29, 30]. Studies have found that educational health interventions can decrease somatic and psychological symptoms in premenopausal and menopausal women; the increased awareness helps them to better understand and cope, making it less likely that women would choose to avoid medical treatment [4, 31].

But the most encompassing approach would be educating young adults who will become menopausal women, or spouses, co-workers, neighbours and close contacts, or physicians of menopausal women. By raising awareness in younger people through interventions and methods they most prefer, such as an open-access website with resources, we can prevent the suffering brought on by menopause in both the woman and her family.

### Limitations

There are a few limitations to this study. A sampling bias is introduced as only university students were chosen to participate in the survey. Furthermore, many participants had likely even received biology and medical teaching, or the survey title may have attracted or dissuaded a certain subset of students from participating. We also can not exclude the possibility that some students were attracted to the survey because of the opportunity to win a $ 50.00 (CAD) gift card and were lesser interested in the topic. Our sample is not representative of the general younger adult population as it overestimates the level of menopause knowledge of the average young adult, making the finding difficult to extrapolate. Nevertheless, it serves as a first step in focusing on this population and assessing their knowledge level. The participants also did not have the option to express their lack of knowledge or uncertainty regarding the survey questions which may lead to answers being guessed, nevertheless, it provides an opportunity for participants to extrapolate from their base knowledge and potentially realize their shortcomings.

### Strength of the study

This study has several strengths. It is the first study of its kind, assessing menopause knowledge in younger adults including all sexes and genders. Although a researcher-made survey was used, it was evidence-based and pilot-tested, and feedback was incorporated from field experts and young adults of both sexes to ensure that the questions were applicable to our target demographic. There was also a large sample size (828 participants) including students from all faculties and backgrounds which allows for a closer approximation of knowledge levels in younger adults receiving post-secondary education. Finally, participants on average reported an increased knowledge confidence post-survey, implying that completion of the survey increases awareness about menopause.

### Knowledge translation

The result from this study provides an opportunity for further studies to examine the younger adult population to truly understand their knowledge of menopause and other women’s health issues. In addition, based on the results we will create free, easy to access, digital resources on menopause and its experience to proactively educate the younger adult population to reduce the stigma around women's health. These resources will arm women and their family members with the knowledge to facilitate intergenerational communication and techniques to support their loved ones, while improving younger women’s experience when they reach menopause, and to ultimately create happier and healthier communities.

## Conclusion

Symptoms such as hot flashes, night sweats, sleep disturbances and vaginal dryness begin during the menopausal transition and persist in post-menopause and for the rest of some women’s lives [32]. These somatic symptoms often present alongside psychological symptoms such as anxiety, depression, and mood fluctuations, reducing the quality of life for these women [33]. In addition, during the menopause transition, women often experience other changes within their family, social and work life which can bring about other stressors. The amalgamation of these changes and the increasing life expectancy of women emphasize the importance of caring for menopausal women who are experiencing a turning point in their lives.

Findings of this study have highlighted that young adults in an academic setting have a general knowledge of menopause and its complications and possess the ability to empathize with and understand menopausal women. Their knowledge level is cursory and is positively affected by their sex (female) and having a connection to menopausal women. Most participants indicated that they were interested in learning more about menopause through electronic resources. According to these findings, young adults are an excellent demographic for an educational intervention as they demonstrate empathy, teachability and an interest in learning more, therefore if we equip them with the right resources and strategies, it can destigmatize women’s health issues and increase support. More specifically, a targeted educational intervention will serve to increase social support and awareness in a cost-effective and sustainable manner, resulting in more open conversations and mitigation of complications, to improve the quality of life in menopausal women and help prepare younger women for their future menopausal journey.

### Supplementary Information

**Additional file 1. **Participant demographic.

**Additional file 2. **All survey questions and corresponding answer options.

## Data Availability

The data pertaining to the current study are available from the corresponding authors in accordance with appropriate data use agreements.
