# Supplementary material for: Assessing young adults' menopause knowledge to increase understanding of symptoms and help improve quality of life for women going through menopause; a student survey
Source: BMC Womens Health. 2023 Sep 15;23:493. doi: 10.1186/s12905-023-02641-4 (PMC10504692; doi:10.1186/s12905-023-02641-4)
Supplement: Supplementary file 1 — Additional file 1. Participant demographic. [file 12905_2023_2641_MOESM1_ESM.docx]

**Additional file 1: Participant demographic**

| **Participant Characteristics** | | **Responses**  N [%] |
| --- | --- | --- |
| ***Age***  (N= 827) | [years (mean±SD)] | 22.1 ± 5.1 |
| ***Biological sex***  (N=828) | Female  Male  Prefer not to answer | 683 [82.5]  138 [16.7]  7 [0.8] |
| ***Gender identity***  (N=828) | Women  Trans women  Man  Trans men  Non-binary  Gender fluid  Two-spirited  Agender  Other  Prefer not to answer | 658 [79.5]  0 [0.0]  137 [16.5]  2 [0.2]  21 [2.5]  2 [0.2]  0 [0.0]  1 [0.1]  3 [0.4]  4 [0.5] |
| ***Ethnicity***  (N= 827) | Biracial or Multiracial  Black (Caribbean, Black African, Black North American, etc.)  Caucasian  East Asian (Chinese, Japanese, Mongolian, Korean, Taiwanese, etc.)  First Nations, Metis or Inuit  Hispanic or Latin American (Mexican, Brazilian, Chilean, etc.)  Middle Eastern or Arab  Pacific Islander  South Asian (Sri Lankan, Bangladeshi, Indian, Pakistani, etc.)  West Asian (Afghan, Iranian, etc.)  Ethnicity not listed here  Prefer not to answer | 35 [4.2]  30 [3.6]  432 [52.2]  111 [13.4]  21 [2.5]  24 [2.9]  15 [1.8]  6 [0.7]  123 [14.9]  2 [0.2]  15 [1.8]  13 [1.6] |
| **Participant University status** | |  |
| ***Student status***  (N=828) | Undergraduate student  Graduate student | 752 [90.8]  76 [9.2] |
| ***Faculty enrolled***  (N= 828) | Agricultural, Life + Environmental Sciences  Alberta School of Business  Arts  Augustana Campus: Fine Arts and Humanities  Augustana Campus: Science  Augustana Campus: Social Sciences  Campus Saint-Jean  Education  Engineering  Kinesiology, Sport, + Recreation  Law  Medicine + Dentistry  Native Studies  Nursing  Pharmacy + Pharmaceutical Sciences  Rehabilitation Medicine  School of Public Health  Science | 46 [5.6]  27 [3.3]  160 [19.3]  6 [0.7]  10 [1.2]  9 [1.1]  14 [1.7]  79 [9.5]  93 [11.2]  33 [4.0]  12 [1.4]  78 [9.4]  3 [0.4]  35 [4.2]  10 [1.2]  10 [1.2]  8 [1.0]  195 [23.6] |
| **Participant relationship and living status** | |  |
| ***Relationship status***  (N= 827) | Single  In a relationship  Married  Separated  Divorced  Widowed  Prefer not to answer | 460 [55.6]  307 [37.1]  48 [5.8]  1 [0.1]  3 [0.4]  0 [0.0]  8 [1.0] |
| ***Living situation***  (N= 827) | Single  Roommate female  Roommate male  Partner female  Partner male  Family | 133 [16.1]  85 [10.3]  23 [2.8]  21 [2.5]  88 [10.6]  477 [57.7] |
| ***Relationship with someone currently in menopause ^a^***  (N= 828) | Mother  Grandmother  Aunt  Other Relative  Other woman ^b^  No relationship and no knowledge of anyone in menopause | 610 [73.7]  214 [25.9]  137 [16.6]  42 [5.1]  234 [28.3]  72 [8.7] |
| ***Close contact with a person in menopause***  (N= 828) | Yes  No | 676 [81.6]  152 [18.4] |

^a^ could include more than one answer

^b^ were described as co-worker, teacher, boss, family friend, friend, neighbor
