# Supplementary material for: Assessing young adults' menopause knowledge to increase understanding of symptoms and help improve quality of life for women going through menopause; a student survey
Source: BMC Womens Health. 2023 Sep 15;23:493. doi: 10.1186/s12905-023-02641-4 (PMC10504692; doi:10.1186/s12905-023-02641-4)
Supplement: Supplementary file 2 — Additional file 2. All survey questions and corresponding answer options. [file 12905_2023_2641_MOESM2_ESM.docx]

**Additional file 2**

All survey questions and corresponding answer options.

| **Question** | **Answer options** |
| --- | --- |
| Are you a student currently enrolled at the University of Alberta? | Yes  No |
| Are you 18 years of age or older? | Yes  No |
| What is your age? |  |
| Are you a graduate or undergraduate student? | Graduate  Undergraduate |
| What is your faculty? | Kinesiology, Sport, + Recreation  Medicine + Dentistry  Nursing  Pharmacy + Pharmaceutical Sciences  School of Public Health  Rehabilitation Medicine  Agricultural, Life + Environmental Sciences Engineering  Science  Alberta School of Business  Arts  Education  Law  Augustana Campus  Campus Saint-Jean  Graduate Studies + Research  Native Studies  St. Joseph's College  St. Stephen's College |
| What is your department or program of study? | Anesthesiology and Pain Medicine  Biochemistry  Biomedical Engineering  Cell Biology  Critical Care  Dentistry and Dental Hygiene  Emergency Medicine  Family Medicine  Laboratory Medicine and Pathology  Medical Genetics  Medical Microbiology and Immunology  Medicine  Obstetrics and Gynecology  Oncology  Ophthalmology  Pediatrics  Pharmacology  Physiology  Psychiatry  Radiology and Diagnostic Imaging  Surgery  Communication Sciences and Disorders  Occupational Therapy  Physical Therapy  Agricultural Food and Nutritional Science  Human Ecology  Renewable Resources  Resource Economics and Environmental Sociology  Biomedical Engineering  Chemical and Materials Engineering  Civil and Environmental Engineering  Electrical and Computer Engineering  Mechanical Engineering  School of Engineering Safety and Risk Management  School of Mining and Petroleum Engineering  Biological Sciences  Chemistry  Computing Science  Earth and Atmospheric Sciences  Mathematical and Statistical Sciences  Physics  Psychology  Accounting and Business Analytics  Finance  Marketing, Business Economics & Law  Strategy, Entrepreneurship & Management  Anthropology  Art & Design  Drama  East Asian Studies  Economics  English and Film Studies  History, Classics & Religion  Linguistics  Media and Technology Studies  Modern Languages and Cultural Studies  Music  Philosophy  Political Science  Psychology  Sociology  Women's & Gender Studies  Educational Policy Studies  Educational Psychology  Elementary Education  School of Library and Information Studies  Secondary Education  Fine Arts and Humanities  Science  Social Sciences |
| Which year of your program will you be entering for the 2021-2022 academic year? | 1  2  3  4  5  6+ |
| Have you completed or participated in post-secondary education prior to your current degree? | Yes  No |
| What was the faculty of your previous post-secondary program? | Kinesiology, Sport, + Recreation  Medicine + Dentistry  Nursing  Pharmacy + Pharmaceutical Sciences  School of Public Health  Rehabilitation Medicine  Agricultural, Life + Environmental Sciences Engineering  Science  Alberta School of Business  Arts  Education  Law  Augustana Campus  Campus Saint-Jean  Graduate Studies + Research  Native Studies  St. Joseph's College  St. Stephen's College |
| What is your biological sex? | Man  Women  Prefer not to answer |
| What is your gender identity? | Woman  Trans woman  Man  Trans man  Non-binary  Gender fluid  Two spirit  Agender  Other  Prefer not to answer |
| What is your self-identified ethnicity? | Asian  Hispanic or Latino  Black or African Canadian  Middle Eastern or North African  First Nations, Metis or Inuit  Pacific Islander  Biracial or multiracial  Ethnicity not listed here  Prefer not to answer |
| What is your relationship status? | Married  In a relationship  Single |
| Which of the following best describes your living situation? | Single  Roommate male  Roommate female  Partner male  Partner female  Family |
| Do you have a close relationship with a woman/women over the age of 50? If yes, please specify the relationship. | Yes, relationship:  No |
| Has anyone in your family undergone or is currently undergoing menopause? If yes, please specify the relationship. | Yes, relationship:  No |
| Do you personally know anyone outside of your family, who has undergone or is currently undergoing menopause? If yes, please specify the relationship. | Yes, relationship:  No |
| On a scale of 1-10 how confident are you in your knowledge about menopause? | 🡨 1, 2, 3, 5, 6, 7, 8, 9, 10 🡪 |
| In a sentence, please describe what menopause is. |  |
| Is experiencing menopause normal or abnormal? | Normal  Abnormal |
| What is the typical age of onset of menopause? | 35-45  45-55  55-65 |
| Can a woman in her 30s undergo menopause? | Yes  No |
| Which of the following is the most appropriate way to confirm menopause status? | Doing yearly blood test for women over 35  Asking family members if the relative in question is behaving differently  Performing an endoscopy  Irregular menses (periods) |
| In a sentence, please answer: when should a woman in menopause seek medical attention? |  |
| Changes in which hormones are mainly associated with menopause? | Follicle stimulating hormone (FSH), Luteinizing hormone (LH), Estrogen  Epinephrine and norepinephrine  Parathyroid hormone (PTH)  Insulin  Melatonin  Adrenocorticotropic hormone (ACTH), cortisol |
| What are the causes of menopause? Select all that apply. | Naturally declining reproductive hormones  Chemotherapy  Lung cancer  Surgical removal of ovaries  Surgical removal of appendix |
| Can lower socioeconomic status contribute to an earlier onset of menopause? | Yes  No |
| Can genetics contribute to an earlier onset of menopause? | Yes  No |
| On a scale of 1-10, how debilitating do you think menopause symptoms are on average for women? | 🡨 1, 2, 3, 5, 6, 7, 8, 9, 10 🡪 |
| On a scale of 1-10, express the level of bodily effect you think menopause has. | 🡨 1, 2, 3, 5, 6, 7, 8, 9, 10 🡪 |
| Which bodily systems can be affected by menopause? Select all that apply. | Reproductive  Cardiovascular  Digestive  Immune  Nervous  Respiratory  Endocrine  Musculoskeletal |
| Do all women experience the same menopause symptoms? | Yes  No |
| Which symptoms are associated with menopause? Select all that apply. | Headaches  Dry eyes  High blood pressure  Indigestion  Hot flashes  Heart disease  Memory loss  Depression  Aching joints  Mood swings  Hair loss |
| Can menopause make women more anxious and irritable? | Yes  No |
| In a menopausal woman, the occurrence of which of the following would require further medical assessment? | 3 consecutive sleepless nights  Panic attacks  Vaginal bleeding 12 months after last period  Decreased libido  5-pound weight gain over a month |
| Which of the following findings are usually NOT associated with menopause? | Decreased libido  Increased energy  Increased facial hair  Decreased height  Increased anxiety |
| Menopause can be a risk factor for which medical conditions? Select all that apply. | Mood disorders  Osteoporosis  Vaginal atrophy  Autoimmune diseases  Cardiovascular diseases |
| Which treatments may be useful for the management of menopause symptoms and long-term effects? Select all that apply. | Vitamin D, Calcium  Aspirin  Pacemaker placed on heart  Nicotine patches  Hormone therapy  Antipsychotic medications  Warm baths  Herbal remedies  Antidepressants |
| Exercise can help improve health and manage many health issues. Which exercises might help address menopause symptoms? Select all that apply. | Walking  Swimming  Yoga  Resistance exercise  Exercises to strengthen pelvic muscles |
| How long do menopause symptoms last? | 3 years after last period  5 years after last period  By age 60  It is variable, sometimes menopause-related symptoms never end |
| Which of the following is a likely scenario occurring due to menopause? | Sumin starting to get oily skin and hair, and acne  Janet forgetting where she put her phone  Dyani having regular menses (periods)  Amira slipping on a patch of ice in her driveway |
| How can you help a woman going through menopause? | Tell her to get a grip  Tell her to take a break  Be kind and supportive  Leave her alone with her emotions |
| Which of the following is the **most sustainable** way to support a woman undergoing menopause? | Buy her an AC unit  Providing social and emotional support  Tell her she will get over it  Keep your distance from her  Buy her a solo vacation to Paris |
| Which of the following should you try to AVOID saying when speaking to a menopausal woman? Select all that apply. | “You can't possibly be going through menopause, you’re too young.”  “How have you been sleeping these days?”  “How are your symptoms today?”  “It’s all in your head.”  “Why are you so moody?” |
| There is an increased risk of experiencing depression during menopause. Which of the following states may contribute to development of depression in a woman undergoing menopause? Select all that apply.  The changes brought on by menopause can place strain on personal relationships. Which of the following could be a contributing factor? Select all that apply. | Unemployment  Increased appetite  Surgical menopause  Lack of social support  Regular exercise  Low libido  Feelings of sadness  Tiredness  Irritability |
| Which of the following is an ineffective way of managing menopausal symptoms? | Exercising  Journaling everyday  Self-isolation to reduce friction with others  Seeking medical help  Using a calendar to keep track of events |
| After completing the survey, on a scale of 1-10 how confident are you in your knowledge about menopause? | 🡨 1, 2, 3, 5, 6, 7, 8, 9, 10 🡪 |
| After completing the survey, on a scale of 1-10 how knowledgeable do you believe your classmates’ and peers’ are on the topic of menopause? | 🡨 1, 2, 3, 5, 6, 7, 8, 9, 10 🡪 |
| Would you be interested in learning more about menopause? If so, then which would be your preferred form? | No  Yes, through physical print outs/ brochures.  Yes, through a lecture delivered by an expert.  Yes, through a website with educational resources. |
